# Supplementary material for: Hops/Tmub1 Heterozygous Mouse Shows Haploinsufficiency Effect in Influencing p53-Mediated Apoptosis
Source: Int J Mol Sci. 2021 Jul 2;22(13):7186. doi: 10.3390/ijms22137186 (PMC8269437; doi:10.3390/ijms22137186)
Supplement: Supplementary file 1 [file ijms-22-07186-s001.zip › ijms-1249630-supplementary.pdf]

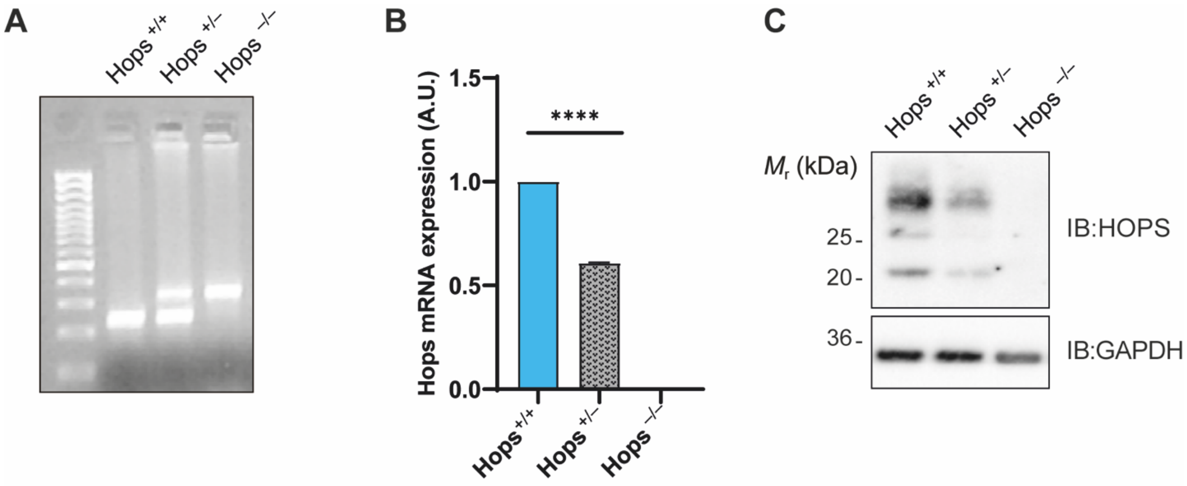

Figure S1. Genotyping of *Hops*$^{+/+}$, *Hops*$^{+/–}$ and *Hops*$^{–/–}$ MEFs. (**A**) PCR result of genotyping by genomic DNA analysis shows a 242-bp band for *Hops*$^{+/+}$ asset, a doublet (242 and 331-bp) for the *Hops*$^{+/–}$ and a 331-bp band for *Hops*$^{–/–}$. (**B**) Genotyping confirmation by real-time PCR for samples as in A. (**C**) Protein extracts from *Hops*$^{+/+}$ and *Hops*$^{+/–}$ and *Hops*$^{–/–}$ MEFs were analysed by Western blot using anti-HOPS antibody and anti-GAPDH antibody as loading control. Values are mean ± SEM, and *p*-values were calculated using ordinary One-Way ANOVA with Dunnett's multiple comparisons test. **** $p < 0.0001$.

Table S1. Primer sequences used in the RT-qPCR assays.

**Target**	**Primer name**	**Sequence (5′→3′)**
β-actin	β-actin F<br>β-actin R	ATTACTGCTCTGGCTCCTA<br>ATCTGCTGGAAGGTGGAC
Bax	Bax F<br>Bax R	GATGAACTGGACAGCAATATGG<br>CGGAAGAAGACCTCTCGG
Hops	Hops F<br>Hops R	GCCTCAGGACACCATTGG<br>CTAGCAGTTGACCTTGGTAGATG
p21	p21 F<br>p21 R	TGTCCAATCCTGGTGATGT<br>CAACTGCTCACTGTCCAC
p53	p53 F<br>p53 R	AACCGCCGACCTATCCTTACC<br>GCACAAACACGAACCTCAAAGC
Noxa	Noxa F<br>Noxa R	GACATAACTGTGGTTCTGG<br>ACTCGTCCTTCAAGTCTG
Puma	Puma F<br>Puma R	CGGCGGAGACAAGAAGAG<br>GAGGAGTCCCATGAAGAGATTG
